# Clinical Challenges in the Management of Hepatic Encephalopathy in Older Patients with Cirrhosis: A Nationwide Italian Physician-Reported Survey

**DOI:** 10.3390/medicina62050955

**Published:** 2026-05-13

**Authors:** Lucia Lapenna, Simone Di Cola, Silvia Nardelli, Manuela Merli

**Affiliations:** Department of Translational and Precision Medicine, Sapienza University of Rome, 00185 Rome, Italy; lucia.lapenna@uniroma1.it (L.L.); simone.dicola@uniroma1.it (S.D.C.); silvia.nardelli@uniroma1.it (S.N.)

**Keywords:** survey, hepatic encephalopathy, older, management, cirrhosis, cognitive impairment

## Abstract

*Background and Objectives*: Hepatic encephalopathy (HE) management becomes particularly challenging in older patients. This study aimed to evaluate the physician-reported diagnostic approaches, therapeutic strategies, and specific challenges in managing HE in older cirrhotic patients across Italy. *Methods*: A nationwide survey was conducted under the aegis of the Italian Association for the Study of the Liver (AISF). Forty-three hepatology centres participated. Data were analyzed using descriptive statistics. *Results*: Participating centers followed over 6000 older patients with cirrhosis, nearly one-third of whom experienced overt HE and/or minimal HE episodes in the previous year. Physicians reported that infections were the most frequently reported precipitating factor, followed by constipation and electrolyte disturbances for OHE. Half of patients with HE (50%; IQR 30.0–50.0%) experienced recurrent episodes, while 22% (IQR 10.0–30.0%) were reported to have persistent HE. In this setting, the diagnosis of HE was often complicated by cognitive decline. Treatment primarily consisted of a combination of lactulose and rifaximin, but adherence was often limited. Caregiver support emerged as a critical element in patient management. The management of comorbidities such as diabetes and chronic kidney disease was a major challenge, and nutritional screening was not routinely implemented across centres. *Conclusions*: This study highlights the need for better multidisciplinary management, improved caregiver support, and more consistent approaches to the diagnosis and treatment of HE in the elderly. The survey also explored how centres approach the differential diagnosis between HE and age-related cognitive disorders, the practical role of caregivers in outpatient management, and the impact of comorbidities, polypharmacy, and nutritional issues on everyday care. Marked heterogeneity emerged in psychometric assessment, multidisciplinary collaboration, and nutritional screening, indicating that several relevant aspects of care remain insufficiently standardized. Overall, the findings suggest that older patients with HE should be regarded as a distinct high-risk subgroup requiring tailored diagnostic pathways and integrated management models.

## 1. Introduction

Hepatic encephalopathy (HE) is a neuropsychiatric syndrome resulting from liver failure and/or portosystemic shunting [1]. It constitutes a common and serious complication of cirrhosis, presenting a wide spectrum of clinical manifestations that range from mild cognitive impairment to coma [2]. Its onset represents a critical turning point in the natural history of cirrhosis and significantly impacts patient prognosis [3], with median survival reduced to approximately 2 years [4] in the general cirrhotic population. In a recent large study by Tapper et al. [5] survival in patients with cirrhosis and HE older than 65 years was associated with median survivals of 0.95 years.

Moreover, this condition is closely linked to a significant deterioration in patient’s health-related quality of life (HRQoL) [6] and causes a considerable caregiver burden [7].

Although the prevalence of HE is difficult to assess, it is estimated to be around 35–45% among patients with cirrhosis [8,9]. Moreover, as the mean age at which cirrhosis is diagnosed is increasing [10] the prevalence of HE in older patients is also rising, driven in part by an ageing population, in part by an increased incidence of metabolic dysfunction-associated steatotic liver disease (MASLD)—typically diagnosed at a more advanced age than other causes of liver disease [11]—and in part by the longer survival of patients with cirrhosis [12]. Notably, from 2004 to 2014, the prevalence of HE among patients aged 65 years and over increased by a relative increase of 1.10% each year, highlighting the growing burden of HE in the older cirrhotic population [10,12].

A recent Italian survey [13] on patients with cirrhosis over 70 years old, although primarily focused on ascites, highlighted the need for specific considerations in the management of decompensated cirrhosis in older adults, which may require adaptations of standard guidelines—largely derived from studies conducted in younger populations.

In older populations, clinical management of HE becomes particularly challenging.

First, older patients frequently present with frailty, sarcopenia, malnutrition or diabetes mellitus, all of which are well-recognized risk factors for the development of OHE [14,15]. Furthermore, they are also more vulnerable to precipitating factors such as electrolyte imbalances, constipation, or infections [16] and often have multiple comorbidities [17] with consequently more limited therapeutic options.

In addition, it is important to emphasise that the cognitive manifestations of HE in older patients can closely mimic those of neurodegenerative conditions such as Alzheimer’s disease, vascular dementia or Parkinson’s disease [18]. The accurate diagnosis of HE in older patients with cirrhosis is frequently complicated by limited awareness of the increasing age at cirrhosis diagnosis and by symptom overlap with other cognitive disorders. The presence of early signs, such as sleep disturbances, impaired concentration, or irritability, are easily overlooked in the absence of targeted psychometric evaluations [19].

Furthermore, fluctuations in attention and extrapyramidal features may misdirect clinicians towards alternative diagnoses, consequently delaying appropriate HE treatment [20]. Beyond these aspects, HE in late life has a particularly disruptive clinical meaning because even relatively subtle cognitive changes may rapidly translate into falls, poor treatment adherence, loss of independence, emergency department access, and institutionalization [21,22]. For this reason, the burden of HE in older adults extends beyond liver-related outcomes and affects the entire care network surrounding the patient.

From a practical standpoint, clinicians are often required to decide whether a fluctuation in mental status reflects reversible HE, delirium related to an intercurrent illness, adverse drug effects, or an underlying neurodegenerative condition. This uncertainty may influence the timeliness of treatment, the decision to hospitalize, and the need for neurological, geriatric, or social support assessment.

In this context, the present nationwide survey investigates the clinical challenges in the management of HE among older cirrhotic patients, aiming to provide real-world insights into diagnostic practices, therapeutic approaches, and disparities across Italian hepatology centres.

## 2. Methods

An online survey was conducted in May 2025 with the support of the Italian Association for the Study of the Liver (AISF), which invited hepatologists from across Italy to voluntarily answer a 46-question questionnaire. To avoid multiple responses from the same institution, hepatologists working in the same centre were explicitly requested—and this was subsequently verified—to submit only one questionnaire per centre. All questions referred to a retrospective review of routine clinical activity performed over the preceding 12 months. Both multiple-choice and open-ended answers were recorded and managed using the REDCap (Research Electronic Data Capture—vers. 8.11.5—© 2026) platform. Collected data were then exported and analysed using descriptive statistics. Each participating centre was asked to report the total number of patients followed at their institution for the condition of interest. Centre-level responses were subsequently aggregated by weighting estimates according to the number of patients reported by each centre, in order to account for differences in centre volume and to better reflect the overall patient population represented in the survey. Specifically, the total number of patients with a given condition reported across all centres was divided by the total number of patients followed across all centres, thereby yielding an overall proportion representative of the surveyed population Percentages referring to the number of patients are reported as median and interquartile range. The questionnaire was conceived to describe routine clinical practice rather than protocol-driven management, and therefore included items addressing not only prevalence estimates and treatment choices, but also barriers to adherence, access to multidisciplinary support, and organizational issues encountered in everyday care. This approach was intended to capture the complexity of managing HE in older adults within heterogeneous real-world settings. Not all individual questionnaire items are presented separately, as survey results were grouped and reported according to the most pertinent findings for clarity and relevance.

The population of interest in the survey consisted of older patients (aged over 70 years) with a diagnosis of cirrhosis and at least one episode of HE within the last 12 months. The questionnaire aimed to explore current clinical approaches to diagnosis, treatment, and care coordination in this specific patient population. The questionnaire was structured around the following thematic areas: patients and general characteristics of participating centres, characteristics of HE, therapeutic management, clinical considerations in the differential diagnosis, caregiver support in patient management, comorbidities, polypharmacy and malnutrition. The complete set of survey questions is provided in the Supplementary Materials.

With respect to HE in the survey, the classic definitions provided by international guidelines were applied [2]. These definitions were established prior to study initiation and were shared with all participating centres to ensure a consistent and homogeneous description across sites. Severity of HE was classified as covert—characterized by minimal or absent clinical signs but detectable abnormalities on neuropsychological and/or neurophysiological testing—or overt (OHE), defined as grade II or higher according to the West Haven criteria. Overt HE was further categorized as episodic, recurrent (more than one episode within a 6-month period), or persistent (no return to baseline neuropsychiatric function between episodes).

The survey was conducted exclusively among healthcare providers and did not involve the collection of identifiable patient data or sensitive personal information. For this reason, in accordance with institutional and national regulations, formal institutional review board or ethics committee approval was deemed unnecessary. Given the descriptive and exploratory nature of the study, no formal hypothesis testing was planned. Instead, the analysis was focused on identifying recurrent patterns in care and highlighting areas of variability that may be especially relevant for the clinical management of older patients with cirrhosis and HE.

## 3. Results

### 3.1. Patients and General Characteristics of Participating Centres

A total of 43 centres participated in the survey. Participants were predominantly gastroenterologists (60%) or internists (30%), half of them from university hospitals (52%). Most of them reported being directly involved in therapeutic decisions (95%). Only 34.9% of respondents were affiliated with a transplant centre, whereas the majority (79.1%) were working in a Gastroenterology department. As for the geographical distribution of the 43 participating centers, 22 were located in Northern Italy, 10 in Central Italy, and 11 in Southern Italy. In total, the centres reported following 6682 older patients with cirrhosis. The prevalence of overt hepatic encephalopathy (OHE, grade 2 or higher according to the West Haven criteria—requiring hospitalization within the past 12 months) in patients with cirrhosis and age over 70 varied, widely across centres, with a median of 20.0% (IQR 13.3–37.5%). In addition, a further 10.0% (IQR 5.0–20.0%) of patients were reported to have had a diagnosis of mild HE (grade 1 according to the West Haven criteria, not requiring hospitalization). Among the patients with OHE, 29% (IQR 13.0–45.0%) died over the past 12 months. The reported causes of death in these patients were infections (47.6%), acute-on-chronic liver failure (ACLF) (21.4%), hepatic coma (4.8%), gastrointestinal bleeding (4.8%), cardiovascular events (4.8%), malignancies (7.1%), and other causes (9.5%). The reported prevalences were calculated considering the number of elderly patients followed by each centre and the number of elderly patients with HE of varying severity, including those who had died, and should be interpreted as estimates inherent to the survey-based design.

### 3.2. Characteristics of HE

**Figure 1:**
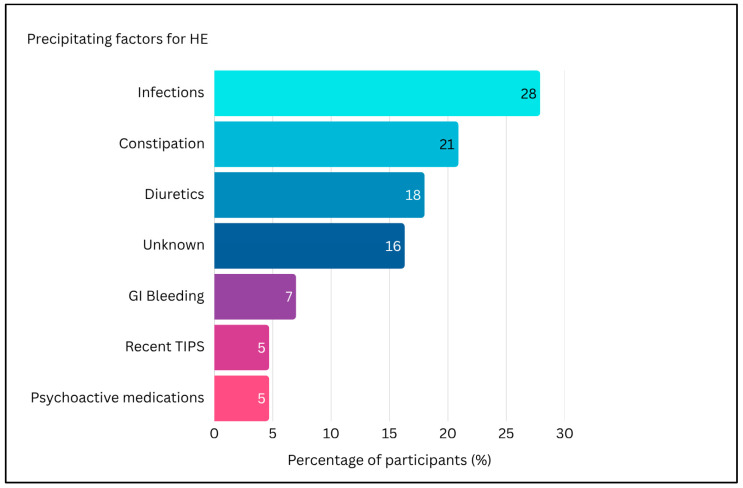
Distribution of precipitating factors for HE episodes in patients with cirrhosis over 70 years old.

**Figure 2:**
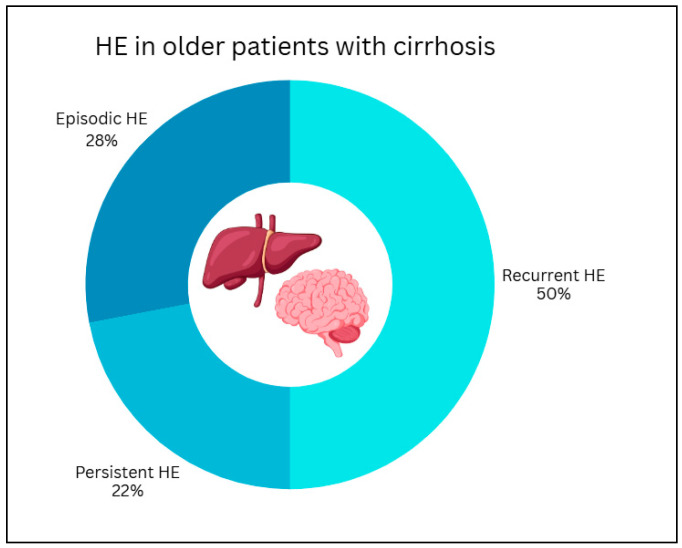
Proportion of older patients with cirrhosis experiencing recurrent HE and persistent HE.

**Figure 3:**
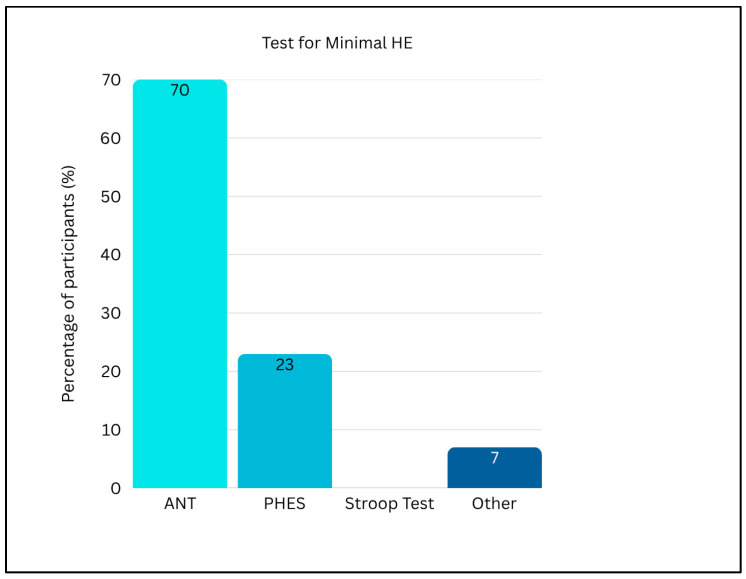
Diagnostic tools used for minimal HE. (ANT: Animal Naming Test; PHES: Psychometric Hepatic Encephalopathy Score).

Participants in the survey were asked to rank the most frequent precipitating factors for HE in older patients. As reported in Figure 1, infections were most reported as the leading cause (27.9%), followed by constipation (20.9%), electrolyte disturbances/dehydration due to diuretics (18.6%), unknown causes (16.3%), gastrointestinal bleeding (4.7%), psychoactive medications (4.7%) and TIPS placement within the previous year (4.7%). Among patients with HE half (50%) (IQR 30.0–50.0%) experienced recurrent HE, while 22% (IQR 10.0–30.0%) were reported to have persistent HE (Figure 2). Regarding the diagnosis of minimal HE (MHE), 86% of participants reported to use psychometric testing in their clinical practice. As shown in Figure 3, the most used tool was the Animal Naming Test (ANT) (70%), followed by the Psychometric Hepatic Encephalopathy Score (PHES) (23%). None of the participants reported using the Stroop Test, while other unspecified methods were indicated in 7% centres.

### 3.3. Therapeutic Management

Participants were asked to analyze the therapeutic approach following the first episode of HE in older patients. The majority of respondents (83.7%) reported prescribing a combination of lactulose and rifaximin as first-line therapy. Reported limitations in adherence to lactulose-based therapy among the older population included difficulties in dose management due to the onset of diarrhoea, which often led to dehydration and significant electrolyte imbalances. Other frequently cited issues were bloating, patient discomfort up to incontinence, and, not uncommonly, problems related to glycaemic control or polypharmacy. Regarding rifaximin, the reported issues were considerably fewer, as it was reported to be generally better tolerated. The main concern highlighted was the pill burden in cases where the 550 mg twice-daily formulation was unavailable, particularly in patients undergoing polypharmacy. Constipation was also mentioned, although with minor clinical relevance. Regarding other responses, 14.3% prescribe lactulose alone as first-line therapy, while 2.3% prescribe rifaximin alone in this population.

### 3.4. Clinical Considerations in the Differential Diagnosis

The survey revealed that around 17% (IQR 10.0–20.0%) of cirrhotic patients over the age of 70 from the participating centres had been diagnosed with cognitive impairment, raising concern about a potentially complex differential diagnosis with OHE and MHE.

When asked about clinical elements that may support a diagnosis of cognitive impairment rather than HE, participants primarily indicated the following: presence of focal neurological deficits, onset of symptoms preceding evidence of advanced liver disease, manifestation of severe episodes of OHE in the absence of precipitating factors; imaging findings consistent with structural brain pathology (such as cortical atrophy, leukoaraiosis, or ischemic lesions), abnormalities in the electroencephalogram (EEG) not related to HE, lack of response to standard therapy for HE or persistently normal ammonia levels.

**Figure 4:**
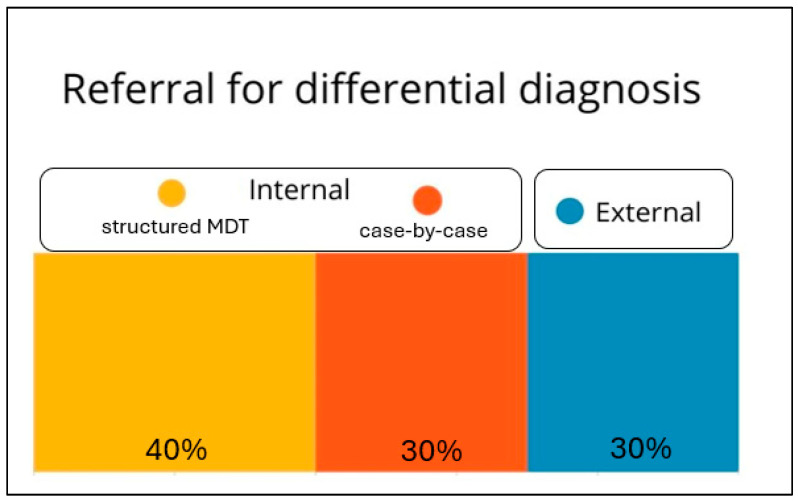
Multidisciplinary approaches to challenging differential diagnoses among participating centers.

In addressing this challenging differential diagnosis, most of the centres (76.7%) rely on multidisciplinary cooperation with neurologists or geriatricians (Figure 4). Approximately 37.2% of centres utilize the Mini-Mental State Examination (MMSE) as a primary screening tool. Referral to local neurological or geriatric evaluation services is practiced by 32.6% of centres, while EEG is employed by 23.3% of centres as an adjunctive diagnostic tool (Figure 3). The vast majority access specialist consultations that are organized on an occasional basis and typically arise from individual collaborations between physicians. The presence of diagnostic uncertainties in this assessment may lead to the misdiagnosis of serious neurological conditions or to HE overtreatment, which was reported to be clinically relevant by 18.6% of the surveyed centres.

### 3.5. Caregiver Support in Patient Management

Among the participants, 35 out of 43 (81.4%) centres reported that only one fourth or less of older patients with HE are independent in managing outpatient visits and home scheduled therapy. At the same time, while all centres unanimously (100%) agreed that a solid caregiver support positively influences the prognosis and quality of life of older patients with HE, only 11.6% rated the actual level of caregiver support as “sufficient.” In contrast, 37.2% described it as “moderate,” 30.2% as “fair,” and as many as 20.9% as “insufficient.” For this reason, approximately 44.2% of centres implement specific caregiver training programs aimed at the early recognition and management of HE symptoms. When the caregiver or the home environment is considered inadequate to support the patient’s care, other assistance plans need to be activated. As a matter of fact, the survey evidenced that more than 30% of patients required external assistance, and about 20% of them were admitted to long-term care facilities or nursing homes.

### 3.6. Comorbidities, Polypharmacy and Malnutrition

In the group of older patients with HE considered in the survey, the most frequently reported comorbidities were type II diabetes, affecting 27.5% (IQR 20.0–38.0%) of patients, and chronic kidney disease, reported in 26% (IQR 15.0–32.0%) of cases. A smaller proportion of patients (8.5%; IQR 2.0–20.0%) had a history of neurovascular events. Regarding chronic therapies known to increase the risk of HE, proton pump inhibitors (PPIs) were reported by 51% of respondents to be used in 25–50% of patients, whereas psychoactive drugs and/or benzodiazepines were prescribed in a lower proportion of cases. For older patients treated with benzodiazepines, 41.9% of those who participate in the survey reported discontinuing their use, 16.3% maintained the therapy unchanged in clinically stable patients, and another 41.9% consulted a neurologist, geriatrician, or psychiatrist to guide treatment decisions. Finally, participants were asked whether diet could play a role in improving the management of older patients with HE. The vast majority (83.7%) replied positively, acknowledging the importance of nutrition in this clinical context. However, only 48.8% of centres performed nutritional screenings on these patients, and just half of the latter refer high-risk individuals for specialised nutritional assessments.

## 4. Discussion

This survey provided an overview of the clinical features, diagnostic challenges, and management of HE in older patients with cirrhosis. The burden of HE is particularly relevant in this population, since the prevalence of cirrhosis is increasing and its clinical presentation is often multifactorial or atypical. A central message emerging from these data is that HE in older adults should be interpreted within a combined hepatologic and geriatric framework. In this setting, liver-related mechanisms interact with frailty, reduced physiological reserve, multimorbidity, and loss of functional autonomy, thereby shaping both the presentation of HE and the resources required for its management.

In our survey, 43 centres provided insights into their clinical experiences with older patients affected by HE. More than 6000 older cirrhotic patients were considered, 20.0% of whom experienced overt HE requiring hospitalization while 10% had minimal. Overall, these data depict an older cirrhotic population with a substantial burden of HE-related decompensation and high short-term mortality, in whom HE appears to occur within a broader context of advanced disease complexity and competing clinical risks.

According to the experience of the participating centres, infections represented the most common precipitating factors of HE in this group of patients, followed by constipation and electrolyte disturbances. The reported pattern suggests that potentially reversible triggers remain highly prevalent in this age group, emphasizing the importance of systematic precipitating-factor assessment during every episode.

Half of the older patients with HE considered in the survey had recurrent episodes of HE, and 22% showed persistent HE. This percentage is considerably higher than the incidence rates of persistent HE reported in the broader cirrhotic population [1]. One possible explanation is that older patients may have a high prevalence of undiagnosed cognitive impairment that is being misclassified as persistent HE, or that a combination of both conditions is present. At the same time, the frequent report of recurrent and persistent forms suggests that, once established, HE often becomes a chronic management problem rather than an isolated event.

Regarding the challenge of differential diagnosis, the main diagnostic difficulty reported by most centres—particularly in older patients with multiple comorbidities—arises from the clinical overlap between HE and other age-related neurodegenerative disorders (e.g., Alzheimer’s disease, vascular dementia, Parkinson’s disease) [9]. In the survey, around 17% of patients with HE also exhibited some degree of cognitive impairment, and accurate diagnosis often required multidisciplinary input, psychometric testing (MMSE), EEG, or neuroimaging. However, there are currently no dedicated diagnostic tests that can reliably assist in this differential diagnosis. Another reported reason to consider a differential diagnosis was the lack of response to therapy with lactulose and rifaximin, suggesting a possible underlying neurological condition. In all such cases, a multidisciplinary team is essential to minimize the risk of misdiagnosis or overtreatment; however, structured multidisciplinary teams were available in only a minority of centres. These findings suggest that differential diagnosis is not an exceptional scenario but a routine component of care in this population, particularly when symptom fluctuations coexist with chronic cognitive decline, neurological comorbidities, or incomplete response to anti-HE therapy.

Therapeutic management predominantly relied on lactulose–rifaximin combination therapy, consistent with current international guidelines [2]. While rifaximin was well tolerated, lactulose adherence was limited by gastrointestinal adverse effects, dehydration, and dosing difficulties, particularly in patients receiving multiple medications [23]. Taken together, the responses show that first-line therapeutic choices were broadly consistent across centres, whereas the main sources of variation concerned treatment tolerability, dose adjustment, and the practical feasibility of maintaining therapy at home in patients with frailty, multimorbidity, or limited autonomy.

Repeated hospitalizations frequently occurred even for potentially manageable episodes, highlighting the lack of community-based resources for outpatient care.

The survey responses consistently portrayed caregiver availability as an essential therapeutic resource rather than a merely accessory element of care. Indeed, caregiver involvement was pivotal, as most patients depended on external support for home-based therapy. Despite widespread recognition of its importance, only a minority of centres provided structured caregiver training, underscoring the need to enhance educational and practical support programs. Structured caregiver education may represent a particularly pragmatic target for improvement, as it could facilitate earlier recognition of precipitating factors, more appropriate titration of bowel-regulating therapy, and more timely contact with healthcare teams before full clinical decompensation occurs.

Comorbidities such as type II diabetes and chronic kidney disease were common, and patients were reported to be frequently exposed to polypharmacy. Chronic use of benzodiazepines, GABAergic agents, opioids, and proton pump inhibitors has been shown to be associated with an increased risk of developing HE [8], while benzodiazepine use is also linked to a higher likelihood of hospitalization due to hepatic encephalopathy. In the surveyed population, a substantial proportion of patients was receiving proton pump inhibitors and benzodiazepines, although benzodiazepines were discontinued in nearly half of the cases. Despite well-established evidence that malnutrition in patients with cirrhosis—particularly in the elderly—can lead to sarcopenia and significantly increase the risk of HE [24], systematic nutritional evaluation, screening, and specialist referral remained inconsistent in the clinical practice. This occurred even though the majority of centers acknowledged the importance of nutritional assessment. The inconsistency in nutritional screening is equally important, since poor oral intake, sarcopenia, and reduced muscle reserve can perpetuate a vicious cycle of weakness, infection risk, functional decline, and recurrent HE. More systematic integration of nutritional assessment into routine hepatology care may therefore provide benefits that extend beyond body composition alone [24].

A further strength of the present survey is that it collected the experience of centres operating in different geographical and organizational contexts, thereby offering a pragmatic snapshot of current national practice rather than outcomes generated under highly selected experimental conditions.

### Limitations

This study has several limitations that should be acknowledged. First, the questionnaire was not formally pilot-tested or validated prior to its use, which may affect the reliability and interpretability of the collected data. In addition, the data are based on retrospective physician-reported estimates rather than patient-level observations, which may have limited their accuracy. However, the complete questionnaire has been included in the Supplementary Materials to enhance transparency and reproducibility. As with all survey-based studies, the results are subject to inherent biases, including response bias, recall bias, selection bias, and social desirability bias. Selection bias may have been influenced by the survey dissemination strategy. Indeed, it was not possible to determine the total number of centres received the questionnaire and therefore the response rate or compare characteristics between participating and non-participating centres. Another aspect to consider is that no formal subgroup analysis between hepatologist responders and other physicians was performed, due to the small number of respondents in the non-hepatologist categories, which limited the robustness of any eventual comparison. Moreover, the single-country setting may limit the generalizability of the findings to other healthcare systems. Another potential limitation to be acknowledged is that the therapeutic options evaluated in the survey were mainly the most standard approaches, while other strategies, such as shunt diameter reduction in patients with post TIPS-HE or branched-chain amino acids supplementation, were not specifically assessed in this elderly population. Furthermore, the lack of stratification by cirrhosis etiology—particularly alcohol-related liver disease, which is highly prevalent in Europe and independently associated with cognitive decline—may have influenced the interpretation of our findings.

Finally, it should be noted that many participants highlighted the significant impact of depressive symptoms on old patients with cirrhosis and HE. However, as there were no specific questions on this topic in the questionnaire, we were unable to provide data on this issue.

## 5. Conclusions

In conclusion, HE in older patients with cirrhosis remains a multifactorial condition characterized by high prevalence, recurrence, and significant diagnostic and therapeutic challenges. In the present survey, some aspects of the management of older patients with HE appear relatively consistent across centres, while other domains remain highly variable, highlighting several relevant practice gaps summarized in Table 1. The findings support the need for multidisciplinary management, individualized treatment strategies, improved community care, and structured caregiver support. Further efforts are needed to strengthen the evidence base. In this context, a possible strategy could be the establishment of a prospective multicenter registry, enabling detailed patient-level characterization of HE in adults older than 70 years. This could provide robust data to inform tailored interventions and ultimately improve clinical outcomes in this vulnerable population.

## Figures and Tables

**Table 1:** Practice gaps in the management of hepatic encephalopathy (HE) in older patients.

Domain of Care	Aspects Relatively Consistent Across Centres	Areas with High Variability	Key Practice Gaps/Priorities for Improvement
Diagnosis of HE	Use of standard clinical criteria for diagnosis of OHE; searching for precipitating factors	screening for minimal HE (MHE); use of psychometric tools	Implementation of systematic and standardized methods for cognitive assessment, especially for MHE detection and differential diagnosis with other neurological disorders frequent in the older patients
Cognitive assessment	Clinical identification of cognitive impairment	Use of validated tools; screening of cognitive status in older adults	Adoption of standardized, age-appropriate cognitive testing protocols with longitudinal monitoring
Nutritional management	General awareness of malnutrition risk in cirrhosis	Nutritional screening in these high-risk patients	Routine nutritional screening and integration of tailored nutritional interventions
Pharmacological treatment	Broad use of guideline-recommended therapies (e.g., lactulose, rifaximin)	Dosage of therapies, adherence monitoring, and treatment escalation strategies	Optimization of treatment adherence and standardized pharmacological approaches for these patients

## Data Availability

The original contributions presented in this study are included in the article/Supplementary Material. Further inquiries can be directed to the corresponding authors.

## Supplementary Materials

The following supporting information can be downloaded at: https://www.mdpi.com/article/10.3390/medicina62050955/s1. Survey of Hepatic Encephalopathy in the Elderly.
