# Supplementary material for: Clinical Challenges in the Management of Hepatic Encephalopathy in Older Patients with Cirrhosis: A Nationwide Italian Physician-Reported Survey
Source: Medicina (Kaunas). 2026 May 13;62(5):955. doi: 10.3390/medicina62050955 (PMC13209091; doi:10.3390/medicina62050955)
Supplement: Supplementary file 1 [file medicina-62-00955-s001.zip › medicina-4243944-supplementary.pdf]

SURVEY OF HEPATIC ENCEPHALOPATHY IN THE ELDERLY

Instructions

Only one physician per centre is requested to complete the questionnaire. Each participating centre is required to review patient medical records prior to completing the questionnaire. Responses should therefore be based on verification of clinical charts. All questions are mandatory except questions no. 1, 2, and 9 (conditional on question 8).

QUESTIONS

1.

In which city do you perform your main clinical or research activity?
Free text response

2.

Please provide your email address for possible correspondence related to this study.
Free text response

3.

How do you define your workplace? (single answer)

- University Hospital
- Non-university Hospital
- Private hospital/clinic
- Other (please specify) ______

4.

How are you involved in therapeutic decision-making for patients with liver disease followed at your centre? (single answer)

- I am the main physician responsible for diagnostic and therapeutic decisions
- I am one of the main physicians responsible, but major decisions are shared with senior colleagues
- I follow patients with liver disease but do not make diagnostic or therapeutic decisions
- Other (please specify) ______

5.

What is your medical specialty? (single answer)

- Gastroenterology
- Internal Medicine
- Infectious Diseases
- Surgery
- Other (please specify) ______

6.

How many patients with a diagnosis of liver cirrhosis are followed at your centre? (single answer)

- 50–100
- 100–300
- 500–1,000
- 1,000

7.

Is your centre also a liver transplant centre?

- Yes
- No

8.

Does your centre have a dedicated Gastroenterology and/or Hepatology ward?

- Yes
- No

9.

If yes, how many beds are available in your ward?
Free numeric response

10.

How many patients with liver cirrhosis aged >70 years are followed at your centre? (Hereafter referred to as "elderly patients")
Free numeric response

11.

Among these elderly patients, how many experienced at least one episode of **overt hepatic encephalopathy** (West Haven grade >2 requiring hospitalization) in the last 12 months?
Free numeric response

12.

Please rank the main precipitating factors of these hepatic encephalopathy episodes by frequency
(1 = most frequent; 7 = least frequent):

- Gastrointestinal bleeding ___
- Constipation ___
- Electrolyte disturbances / diuretic-induced dehydration ___
- Infections ___
- Use of psychoactive drugs ___
- TIPS placement within the last year ___
- Unknown ___

13.

Among these elderly patients, how many were diagnosed with **mild hepatic encephalopathy** (West Haven grade 1, not requiring hospitalization)?
Free numeric response

14.

Among these elderly patients, how many were diagnosed with **recurrent hepatic encephalopathy**
(more than one episode within 6 months according to EASL guidelines)?
Free numeric response

15.

Among these elderly patients, how many were diagnosed with **persistent hepatic encephalopathy**
(persistent cognitive impairment despite resolution of the acute episode)?
Free numeric response

16.

After the first episode of hepatic encephalopathy, which therapy do you most frequently prescribe in elderly patients? (single answer)

- Non-absorbable disaccharides
- Rifaximin
- Lactulose + rifaximin
- Dietary management only (low-protein diet)
- No treatment

17.

What do you consider the main limitations in prescribing non-absorbable disaccharides in elderly patients?
Multiple choice and/or free text

18.

What do you consider the main limitations in prescribing rifaximin in elderly patients?
Multiple choice and/or free text

19.

In your clinical practice, do you use psychometric tests (e.g. Mini-Mental State Examination) for the diagnosis of minimal hepatic encephalopathy?

- Yes
- No

20.

If yes, which ones? (multiple answers)

- PHES
- ANT
- Stroop test
- Other

21.

What percentage of patients aged >70 years with liver cirrhosis followed at your centre have a diagnosis of cognitive impairment?
Free numeric response

22.

Which elements make you suspect cognitive impairment rather than minimal hepatic encephalopathy?
Multiple choice and/or free text

23.

How do you approach the differential diagnosis between cognitive impairment and hepatic encephalopathy in patients with cirrhosis? (multiple answers)

- Use of Mini-Mental tests
- Referral to outpatient neurological/geriatric evaluation
- Internal collaboration with neurologists/geriatricians
- Use of electroencephalography
- No specific approach
- Other (please specify)

24.

Do you think cognitive impairment may lead to overtreatment for hepatic encephalopathy in elderly patients with cirrhosis? (single answer)

- Yes, definitely
- Never
- Yes, partially
- Rarely

25.

Do you have access to a multidisciplinary team (e.g. gastroenterologist/hepatologist, neurologist/geriatrician, nutritionist, radiologist) for the management of these patients?

- Yes
- No

26.

How would you rate the level of caregiver support for elderly patients with hepatic encephalopathy? (single answer)

- Adequate
- Fair
- Moderate
- Inadequate

27.

Do you think adequate caregiver support can improve prognosis and quality of life in elderly patients with hepatic encephalopathy? (single answer)

- Yes
- No
- Marginally

28.

Do you implement specific caregiver training programs for early recognition and management of hepatic encephalopathy symptoms? (single answer)

- Yes
- No
- Rarely
- I would like to, but I lack time and resources

29.

How many elderly patients required activation of home care services (CAD)?
Free numeric response

30.

How many elderly patients are currently admitted to long-term care facilities or enrolled in home/residential hospice care?
Free numeric response

31.

What percentage of elderly patients with hepatic encephalopathy are independent in managing outpatient visits and home therapy? (single answer)

- 0–25%
- 25–50%
- 50–75%
- 75–100%

32.

What percentage of elderly patients with hepatic encephalopathy are on chronic proton pump inhibitor therapy? (single answer)

- 0–25%
- 25–50%
- 50–75%
- 75–100%

33.

What percentage of elderly patients with hepatic encephalopathy are on chronic psychoactive drugs and/or benzodiazepines? (single answer)

- 0–25%
- 25–50%
- 50–75%
- 75–100%

34.

When you evaluate a patient on benzodiazepines with a history of hepatic encephalopathy, how do you usually manage therapy? (single answer)

- Discontinue benzodiazepines
- Maintain therapy if clinical condition is stable
- Consult a neurologist/geriatrician/psychiatrist
- Unsure

35.

How many elderly patients with hepatic encephalopathy died in the last 12 months?
Free numeric response

36.

What was the main cause of death in these elderly patients over the last 12 months? (single answer)

- Infections
- ACLF
- Hepatic coma
- Bleeding
- Cardiovascular events
- Malignancies
- Other (please specify) ______

37.

Do you believe diet plays a role in improving management of elderly patients with hepatic encephalopathy? (single answer)

- Yes
- No
- Marginally

38.

Do you routinely perform nutritional screening in elderly patients with hepatic encephalopathy? (single answer)

- Yes
- No
- Sometimes

39.

Do you refer patients at risk of malnutrition for nutritional assessment and intervention? (single answer)

- Yes
- No
- Sometimes
- I would like to, but I lack resources

40.

How many elderly patients with hepatic encephalopathy have type II diabetes mellitus?
Free numeric response

41.

How many elderly patients with hepatic encephalopathy have chronic kidney disease?
Free numeric response

42.

Among these, how many have stage IV–V disease or are on chronic dialysis?
Free numeric response

43.

How many elderly patients with hepatic encephalopathy have a history of cerebrovascular events?
Free numeric response

44.

Do you have any additional comments on this topic not addressed by the previous questions?
Multiple choice and/or free text

45.

From your perspective, please summarize the main challenges in managing these patients.
Multiple choice and/or free text

46.

What suggestions would you provide to overcome and improve the management of hepatic encephalopathy in elderly patients?
Multiple choice and/or free text
